# Pediatric Ovarian Growing Teratoma Syndrome

**DOI:** 10.1155/2017/3074240

**Published:** 2017-06-01

**Authors:** Rebecca M. Rentea, Aaron Varghese, Atif Ahmed, Alexander Kats, Michelle Manalang, Tazim Dowlut-McElroy, Richard J. Hendrickson

**Affiliations:** ^1^Department of Surgery, Children's Mercy Hospital, Kansas City, MO, USA; ^2^Department of Obstetrics and Gynecology, University of Missouri, Kansas City, Kansas City, MO, USA; ^3^Department of Pathology, Children's Mercy Hospital, Kansas City, MO, USA; ^4^Department of Hematology, Oncology, and Bone Marrow Transplantation, Children's Mercy Hospital, Kansas City, MO, USA

## Abstract

Ovarian immature teratoma is a germ cell tumor that comprises less than 1% of ovarian cancers and is treated with surgical debulking and chemotherapy depending on stage. Growing teratoma syndrome (GTS) is the phenomenon of the growth of mature teratoma elements with normal tumor markers during or following chemotherapy for treatment of a malignant germ cell tumor. These tumors are associated with significant morbidity and mortality due to invasive and compressive growth as well as potential for malignant transformation. Current treatment modality is surgical resection. We discuss a 12-year-old female who presented following resection of a pure ovarian immature teratoma (grade 3, FIGO stage IIIC). Following chemotherapy and resection of a pelvic/liver recurrence demonstrating mature teratoma, she underwent molecular genetics based chemotherapeutic treatment. No standardized management protocol has been established for the treatment of GTS. The effect of chemotherapeutic agents for decreasing the volume of and prevention of expansion is unknown. We review in detail the history, diagnostic algorithm, and previous reported pediatric cases as well as treatment options for pediatric patients with GTS.

## 1. Introduction

Growing teratoma syndrome (GTS) is thought to be the progression of immature to mature teratoma during or following chemotherapy. GTS requires three criteria: normalization of previously elevated tumor markers (AFP or *β*HCG), enlargement of the primary tumor, or finding of new tumor mass and only mature teratoma elements on pathologic examination. The incidence of GTS is 12% of ovarian germ cell tumors (GCT) occurring in young adults and adolescents [1–3]. GTS is most likely to occur if mature teratoma elements are found in the primary tumor, and there is no reduction in tumor size following chemotherapy or incomplete resection of the primary tumor [4].

## 2. Case

**Figure 1:**
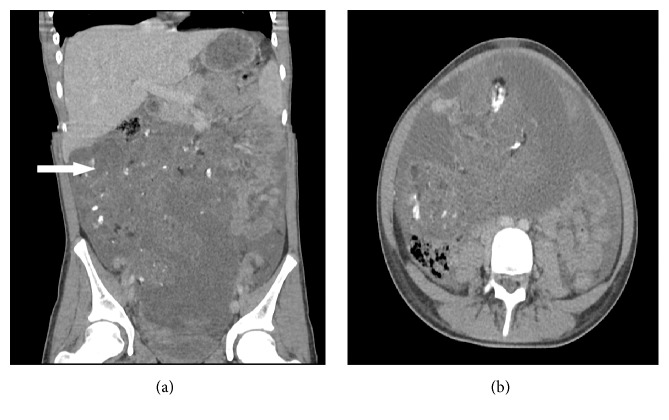
Initial imaging computed tomography of the abdomen and pelvis demonstrating a large pelvic tumor (arrow).

**Figure 2:**
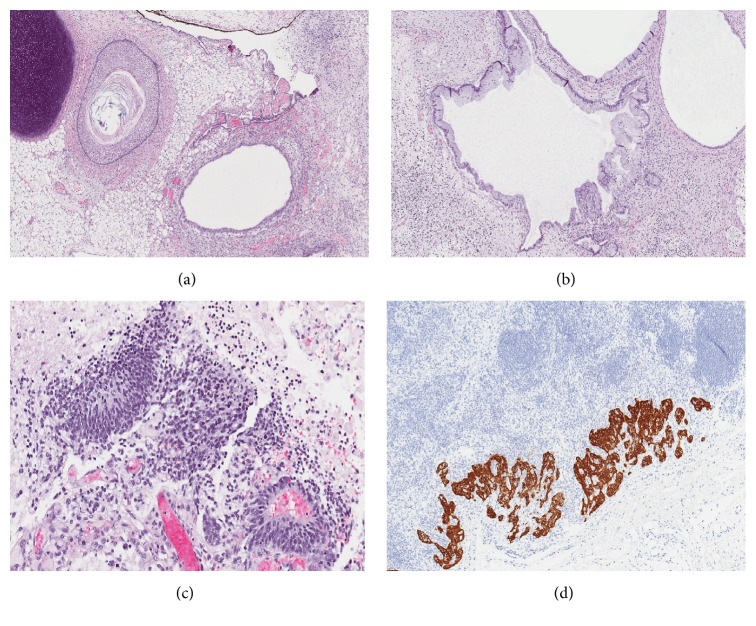
Initial ovarian teratoma with immature elements. (a) Sections of immature teratoma revealed a neoplasm composed of heterogenous elements that included a dermoid cyst, fibroadipose tissue, cartilage, pigmented choroid, and glial tissue (H&E ×100). (b) Cystically dilated glands with enteric differentiation were present (H&E ×100), (c) as well as several foci of immature neuroepithelium (H&E ×200). (d) Immunohistochemical staining with glial fibrillary acid protein (GFAP) reveled teratoma deposits in the periaortic lymph node (GFAP ×200).

A 12-year-old female patient presented from a referring institution in November 2015 for evaluation of 1-month history of an enlarging abdominal mass. The patient reported a 4.5 kg weight loss with increased lethargy. The physical examination was normal except for a large abdominal solid mass. Pelvic CT (Figure 1) revealed a large pelvic mass. Tumor markers were significant for alpha fetoprotein (AFP) 656 ng/mL (nml ≤ 7 ng/mL), CA-125 401 U/mL (nml < 35 U/mL), and CEA 6.7 ng/mL (nml ≤ 3.5 ng/mL). Inhibin A/B and *β*-HCG levels were normal. She underwent an exploratory laparotomy, right salpingoophorectomy, omentectomy, peritoneal washings, diaphragmatic/peritoneal/pelvic biopsies, and periaortic lymph node biopsy. Intraoperative findings were significant for a distinct right ovarian mass with evidence of prior rupture. Ascites was noted and aspirated. There was peritoneal seeding along the pelvis and right pericolic gutter and along the right hemidiaphragm. Gross residual disease remained consisting mostly of the peritoneal seeding despite removal of all gross abdominal disease. The cytology examination of the peritoneal fluid demonstrated no malignant cells. Histologically, all the pathology specimens returned with high grade immature teratoma (Figure 2). She was diagnosed as grade 3, FIGO stage IIIC. Chromosome analysis returned trisomy 3 in four of her autosomal cells, consistent with the diagnosis of immature teratoma. Since there is currently no “standard of care” for pediatric grade immature teratoma FIGO stage IIIC, we elected to follow tumor markers closely. Following tumor markers, her AFP fell by approximately 50% every week almost normalizing over the course of three months from diagnosis (from 656 ng/mL to 9.78 ng/mL). In January 2016, repeat tumor marker labs demonstrated that AFP levels increased to 28.3 ng/mL and CA-125/*β*-HCG were within normal limits (18 units/mL CA-125, <3 units/mL). Repeat CT of chest, abdomen, and pelvis revealed that, in the abdomen and pelvis, she now had enlarged peritoneal implants, as large as 3.5 × 1.5 cm, but no other masses, and, in the chest, she had a 1.3 × 2 × 0.3 cm left anterior pleural nodule, as well as bilateral diaphragmatic lesions, 2.5 × 2.1 × 1.8 cm on right and 2.1 × 3 × 1.5 cm on left. She subsequently underwent chemotherapy with bleomycin, etoposide, and cisplatin (BEP) between February and May 2016 for high risk malignant ovarian germ cell tumor. After 4 cycles, her AFP was 0.84 ng/mL.

**Figure 3:**
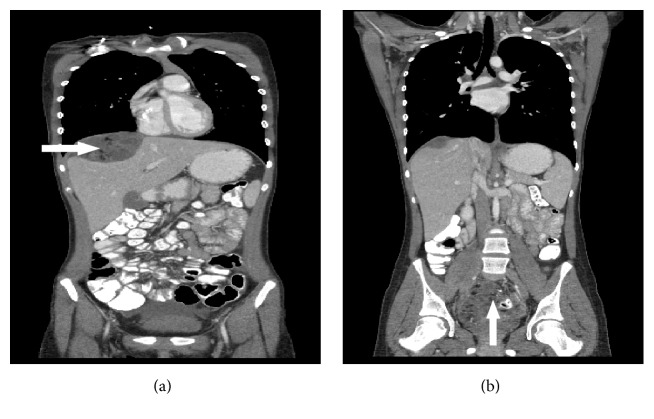
Computed tomography abdomen and pelvis follow-up imaging at 8 months. (a) Large mass occupying the right upper abdominal cavity, revealing multiple new masses containing cystic and necrotic elements surrounding the liver (arrow). Because of tumor growth, the giant mass had compressed the liver parenchyma. (b) Pelvic tumor is demonstrated as well (arrow).

**Figure 4:**
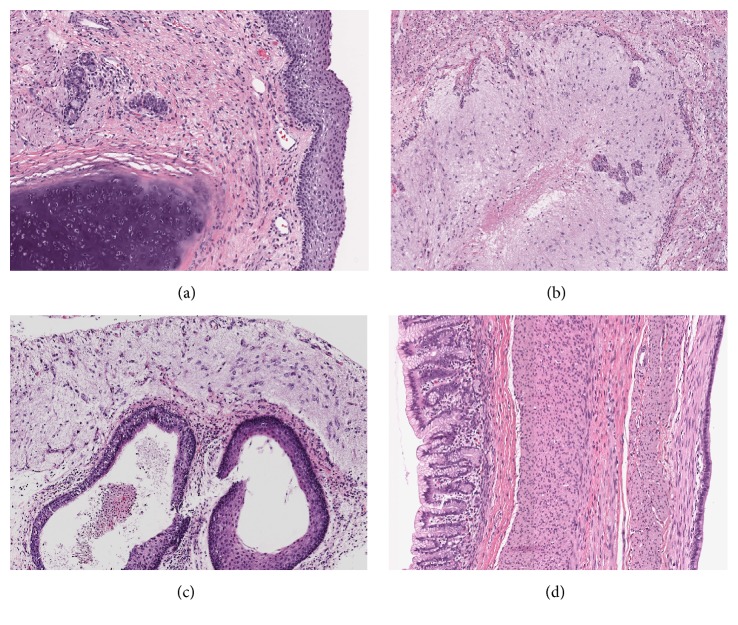
Histology of multiple pelvic lesions following repeat laparotomy demonstrated neoplasms composed of derivatives of all three germ layers, namely, ectoderm, mesoderm, and endoderm consistent with mature teratoma. (a) Mature cartilage and squamous epithelium (H&E ×100). (b) Mature neuroglia and vascular proliferation (H&E ×200). (c) Cysts lined with mature squamous and ciliated columnar respiratory epithelium and overlying mature neuroglia (H&E ×200). (d) Large cyst wall with gastric mucinous epithelium, bundles of smooth muscle, and adjacent cysts lining of the ciliated respiratory epithelium (H&E ×200).

After 4 cycles, she underwent interval radiologic evaluation in preparation for second look surgery. The CT scan of the chest, abdomen, and pelvis in May 2016 revealed a significant increase in the size of the thoracic masses, as well as the size and a number of abdominal and pelvic masses (Figure 3). The largest mass was 7.9 × 7.7 × 8.7 cm in the rectouterine pouch with evidence of fat and calcification. There was also a 4.4 × 7.2 × 6.6 cm peritoneal implant superior to the liver that also had similar visual findings. There were also findings of bilateral cardiophrenic masses, with the largest measuring 2.1 × 1.3 × 1.1 cm. In June 2016, she underwent a second debulking procedure consisting of complete infracolic omentectomy, para-aortic lymph node dissection, hepatic mass resection, and pelvic tumor removal on the right side. Gross residual disease remained in her abdomen and pelvis, since she had innumerous peritoneal studding, and thus her thoracic tumors were not removed. A total of 5 pelvic masses and 3 liver masses were excised, and pathologic analysis indicated mature teratoma (Figure 4). At the time of surgery her liver masses were found to compress the parenchyma of the liver and were not invasive.

Due to the massive growth of tumor in 4 months, as well as recently undergoing 2 major surgical debulkings, and still being unable to completely resect her tumors, medical treatment was discussed with the patient and her parents. In an attempt to prevent regrowth of her tumors, in August 2016, the patient was started on experimental protocol #009 of the Neuroblastoma and Medulloblastoma Translational Research Consortium (NMTRC). This treatment regimen consists of temozolomide 40 mg/m^2^ PO daily (days 1–28), tretinoin 25 mg/m^2^/day PO BID (days 1–14), and sorafenib 150 mg/m^2^ PO BID (days 1–28). Thalidomide was started during cycle 2. This protocol was chosen as this study tests the feasibility of experimental technologies to determine a tumor's molecular makeup. This technology includes a genomic report based on DNA exomes and RNA sequencing that will be used to discover new ways to understand cancers and potentially predict the best treatments for patients with cancer in the future. Prior to the experimental therapeutics, a CT scan was obtained to evaluate disease progression. She is currently without new tumor growths.

## 3. Discussion

Immature ovarian teratoma is a rare germ cell neoplasm that comprises <1% of ovarian teratomas [5]. The immature ovarian teratoma must be distinguished from the common benign mature teratoma (dermoid cyst). The difference between the malignant and benign tumors is the presence of immature components most predominantly neuroectodermal, such as neural and glial cells [6]. GCT typically present within the first two decades of life. About 60–70% of malignant germ cell ovarian tumors are diagnosed as FIGO stage I. Stage IA dysgerminoma and grade-I immature teratoma without ascites are treated effectively with surgery alone. In adults, higher staging requires at least 3 courses of BEP (bleomycin, etoposide, and cisplatin) chemotherapy, but children may not need postoperative chemotherapy [7, 8]. The Children's Cancer Group (CCG) recommends obtaining peritoneal fluid, the removal of tumor without compromising surroundings structures, sparing the fallopian tube if not adherent to the tumor, examination of omentum with removal of suspicious areas, and palpation of pelvic and para-aortic lymph nodes with biopsy in case of abnormality in malignant ovarian tumors. Thus, the relative risk of incomplete staging is higher for pediatric general surgeons [9]. Stratifying grade 3 tumors by stage estimated 5-year event-free survival for patients with grade 3, stage I/II disease was 0.92 (0.72–0.98) for estimated proportion of event-free survival, whereas it was 0.52 (0.22–0.75) for grade 3, stage III patients (*p* = 0.005) [10]. The overall survival for all grade 3 patients, regardless of stage, was 100%. Neither the age at diagnosis nor the AFP level was related significantly to the risk of relapse. The administration of postoperative chemotherapy did not decrease the risk of relapse in the pediatric cohort [10]. Similarly to our study, other pediatric studies have shown no benefit of adjuvant chemotherapy postoperatively in the management of ovarian immature teratomas. In a nonrandomized study by Göbel et al. [11], 76 patients were treated by surgery alone, and 40 patients received adjuvant chemotherapy.

GTS was first introduced by DiSia et al. in 1977 and was called “chemotherapeutic retroconversion” (CR) [12] while Logothetis et al. in 1982 coined the term GTS [13]. Amsalem et al. concluded that these two findings were likely the same event [6]. Currently the pathologic mechanism is thought to arise from either malignant cells differentiation into mature elements or the fact that chemotherapy destroys immature cells leaving mature cells to survive [14].

Ovarian GTS has been described in 101 published English literature cases [15, 16]. Most of the patients had abdominal symptoms, such as abdominal pain and distension when they first sought evaluation. The median age for the diagnosis of primary immature teratoma was 22 years (range 4–48 years, *n* = 56) [15]. Many cases of GTS metastasis spread in the peritoneal cavity and tend to occur in the pelvis, peritoneum, or retroperitoneum but have been located in other places such as the liver, pineal gland, and mediastinal/cervical lymph nodes [7, 14, 17, 18]. A single case report described spread via both the lymphogenous and hematogenous route in the same patient [18]. GTS tumors have a growth rate of 0.5 to 0.7 cm/month and a volume increase of 9.2 to 12.9 cm^3^/month [19, 20].

Recurrent tumors following chemotherapy for germ cell tumors should be resected to confirm diagnosis, relieve impending/possible obstruction, and prevent future malignant transformation [6]. GTS has a high recurrence rate between 72% and 83% with partial resection versus 0% and 12.7% in those who undergo complete resections [20, 21]. Laparoscopy can be utilized as a diagnostic and therapeutic approach in cases of questionable or limited GTS [16].

GTS histology is comprised of mature teratoma elements, but morbidity and mortality arise from its local expansion and the potential for malignant degeneration [20, 22, 23]. There have been cases of safe IVC resection secondary to production of collateral vasculature [24]. Approximately 3% of GTS cases are associated with malignant transformation including adenocarcinoma, sarcoma [25, 26], or primitive neuroectodermal tumors [4]. GTS nodules can appear at any stage during or after chemotherapy, up to 8 years posttreatment, with an average interval of 8 months [22, 27, 28]. Therefore, regular follow-up contributes to early detection, diagnoses, and treatment.

A rare form of tumor spread can be associated with GTS known as Gliomatosis Peritonei (GP). This tissue is composed of mature glial tissue and can be located in the peritoneal cavity and omentum in patients with ovarian teratoma. GP is often mixed with GTS series despite this being a separate entity [13] because it is defined by pure mature glial tumor tissue in the peritoneum [29–31] and is encountered in a variety of clinical scenarios [29, 32, 33]. For example, GP can be found in children with ventriculoperitoneal shunts where it is thought that the cerebrospinal fluid's neural growth factor induces glial differentiation [34, 35]. GP ultimately acts as a prognostic factor for patients with immature ovarian teratoma as those with GP have a shorter recurrence-free survival (2-year recurrence-free survival rates were 59.3 if with GP versus 96.3% without) [36]. In pediatric patients, larger studies are needed to confirm this and it has not been thought to be true, and the presence of GP does not upstage them.

**Figure 5:**
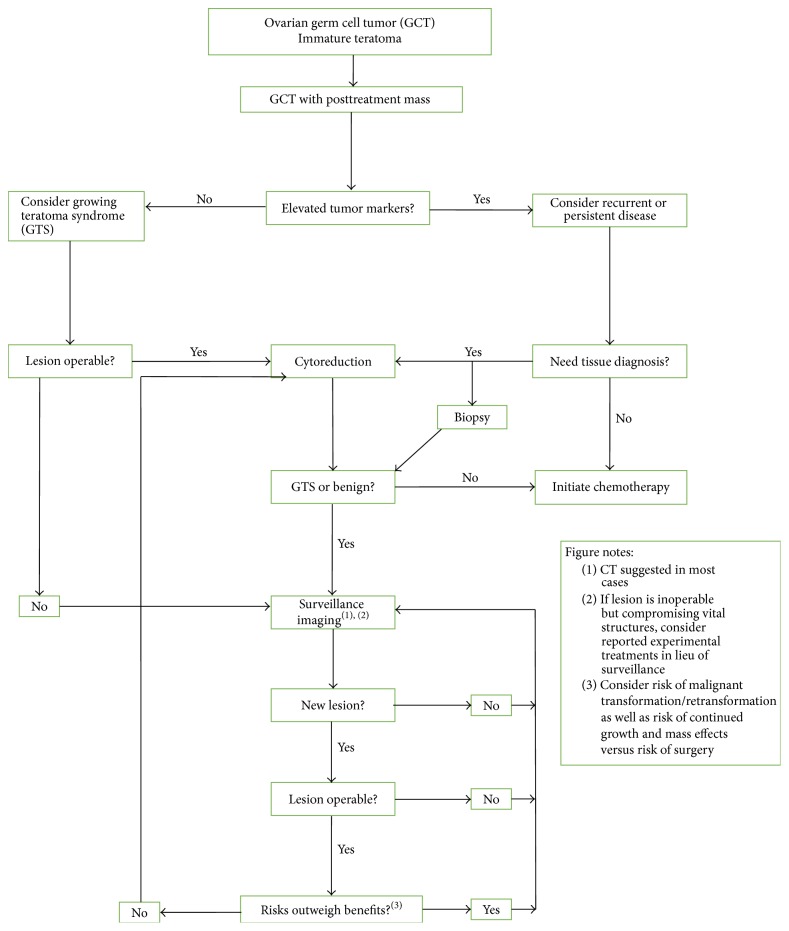
Algorithm suggested for management of pediatric female patients with ovarian germ cell tumors following initial resection of immature teratoma and completion of therapy. GCT, germ cell tumor; GTS, growing teratoma syndrome. Modified from Byrd et al. with permission.

No standardized management protocol has been established, but one has been set out by Byrd et al. [1, 37] (Figure 5). A key point regarding patients with GTS is regular follow-up as the majority of GTS is found on routine follow-up and presents without symptoms [21, 37]. MR imaging and CT are the preferred modalities [38]. CT scan may show a low-density cystic lesion or an increase in the cystic component of the mass suggestive of teratomatous element [39]. CT imaging typically shows radiographic maturation including increased density, better circumscribed margins and onset of internal calcifications, an amalgamation of fat, and solid/cystic components [21, 40]. A downside of CT scans is that it underestimates tumor masses smaller than 1-2 cm particularity within the mesentery and omentum when closely related to small bowel loops in the absence of ascites [39]. FDG-PET as a modality for detection of multiple masses associated with GTS has low utility for diagnosis as the GTS lesions do not uniformly appear as hypermetabolic tissue such as brain and thyroid [41]. Ultrasonographic surveillance is made difficult by the variable appearance of these lesions and it is also less sensitive for fat than other modalities [42]; but it contains no radiation for the patient.

Adjuvant chemotherapy with bleomycin, etopside, and cisplatin is recommended for patients when diagnosed with immature teratoma following primary surgery. Palbociclib, a CDK4/6 inhibitor (PD0332991), is reported that it can stabilize the vascularization of the tumor in pediatric patients with an intracranial teratoma [43]. It is a selective reversible inhibitor of cyclin-dependent kinases (CDK) 4 and 6. Inhibition of CDK 4/6 blocks DNA synthesis by prohibiting progression of the cell cycle from G1 to S phase [43]. The main side effect appears to be reversible neutropenia [44]. It has been especially effective in pRB-expressing teratomas (retinoblastoma protein) [45]. While further investigation of the use of Palbociclib in patients with GTS should be carried out [43], the number of major disease-related clinical events decreases with use of Palbociclib as did the median number of progression-free survival months for unresectable mature teratoma [46]. Inoperable metastases have been known to be treated with bevacizumab and cyclin-dependent kinase inhibitors [47], with tumor marker AFP usually returned to within the normal range [48, 49]. Interferon-alpha has been successfully used in unresectable tumors to stop their growth for a prolonged period of time based on its antiproliferative and antiangiogenetic abilities and by its immune modulatory effect [50–53], but tumors while stabilized can stay the same size [54, 55].

Surgical approaches stress fertility sparing surgery as recommended for women of childbearing age. Successful pregnancy following development of GTS has been reported indicating necessity of fertility sparing approaches [56]. The treatment of choice during childbearing age for immature teratoma is composed of unilateral oophorectomy and in the case of metastatic disease postoperative chemotherapy. Preservation of fertility is a major concern for young patients undergoing surgical bulking and chemotherapy. Low antimullerian hormone (AMH) levels are associated with a higher incidence of posttreatment amenorrhea [57]. Options for fertility preservation include embryo cryotherapy, oocyte cryopreservation, ovarian autotransplantation, in vitro maturation (IVM), and oophoropexy [58]. New innovations in cryotherapy, such as vitrification, allow for rapid cooling of tissue and decrease the risk of intracellular crystallization and damage.

## 4. Conclusion

In the setting of immature ovarian teratoma treated with chemotherapy, a rapid growth rate in the presence of normalized serum tumor markers should raise suspicion of GTS [20]. No effective medical treatment of GTS exists due to its unresponsiveness to chemotherapy or radiotherapy. Treatment is currently excision of GTS lesions and close monitoring with imaging and tumor markers. Some trials indicate various treatments such as CDK inhibitors for inoperable disease or stabilizing growth of recurrent lesions. GTS has an overall good prognosis with few reported deaths.
